# Anti-*Toxoplasma gondii* effect of lupane-type triterpenes from the bark of black alder (*Alnus glutinosa*) and identification of a potential target by reverse docking

**DOI:** 10.1051/parasite/2022008

**Published:** 2022-02-10

**Authors:** Pierre Darme, Sandie Escotte-Binet, Julien Cordonnier, Simon Remy, Jane Hubert, Charlotte Sayagh, Nicolas Borie, Isabelle Villena, Laurence Voutquenne-Nazabadioko, Manuel Dauchez, Stéphanie Baud, Jean-Hugues Renault, Dominique Aubert

**Affiliations:** 1 Université de Reims Champagne Ardenne, ESCAPE EA 7510 51097 Reims France; 2 Université de Reims Champagne Ardenne, CNRS, ICMR 7312 51097 Reims France; 3 NatExplore SAS 51140 Prouilly France; 4 Université de Reims Champagne Ardenne, MEDyC UMR 7369 51093 Reims France; 5 Université de Reims Champagne Ardenne, P3 M 51097 Reims France

**Keywords:** *Toxoplasma gondii*, *Alnus glutinosa*, triterpene, Betulone, Inverse docking, Target hypothesis

## Abstract

Toxoplasmosis is a worldwide parasitosis that is generally benign. The infestation may pose a risk to immunocompromized patients and to fetuses when pregnant women have recently seroconverted. Current treatments have numerous side effects and chemoresistance is emerging, hence the need to find new anti-*Toxoplasma gondii* substances. This study focuses on the antiparasitic potential of lupane-type pentacyclic triterpenes isolated from the bark of black alder (*Alnus glutinosa*), as well as the hypothesis of their macromolecular target by an original method of reverse docking. Among the isolated triterpenes, betulone was the most active compound with an IC_50_ of 2.7 ± 1.2 μM, a CC_50_ greater than 80 μM, and a selectivity index of over 29.6. An additional study of the anti-*T. gondii* potential of commercially available compounds (betulonic acid methyl ester and betulonic acid) showed the important role of the C3 ketone function and the C28 oxidation level on the lupane-type triterpene in the antiparasitic activity since their IC_50_ and CC_50_ were similar to that of betulone. Finally, the most active compounds were subjected to the AMIDE reverse docking workflow. A dataset of 87 *T. gondii* proteins from the Protein Data Bank was created. It identified calcium-dependent protein kinase CDPK3 as the most likely target of betulin derivatives.

## Abstract (fr)

La toxoplasmose est une parasitose mondiale, généralement bénigne. Les personnes à risque sont les patients immunodéprimés et les fœtus chez les femmes enceintes nouvellement séroconverties. Les traitements actuels ont de nombreux effets secondaires et des phénomènes de chimiorésistance apparaissent, d’où la nécessité de trouver de nouvelles substances actives contre *T. gondii*. Cette étude porte sur le potentiel antiparasitaire des triterpènes pentacycliques de type lupane isolés de l’écorce de l’aulne glutineux (*Alnus glutinosa*) et formule une hypothèse quant à leur cible protéique par l’utilisation d’une méthode originale de *docking* inverse. Parmi les triterpènes isolés, la bétulone s’est révélée être la plus active avec une CI_50_ de 2,7 μM ± 1,2 μM, une CC_50_ supérieure à 80 μM et un indice de sélectivité supérieur à 29,6. L’étude complémentaire du potentiel anti-*T. gondii* de composés disponibles commercialement et analogues à la bétulone (acide bétulonique et methyl ester de l’acide bétulonique) a montré le rôle important de la fonction cétone en C3 et du degré d’oxydation de la position 28 du squelette triterpénique de type lupane dans l’activité antiparasitaire puisque leurs CI_50_ et CC_50_ étaient similaires aux valeurs rencontrées pour la bétulone. Enfin, les composés les plus actifs ont été soumis au flux de travail de *docking* inverse d’AMIDE. Un ensemble de 87 protéines de *T. gondii* de la *Protein Data Bank* a été créé. La protéine kinase calcium dépendante CDPK3 a été identifiée comme la cible la plus probable des dérivés de la bétuline.

## Introduction

*Toxoplasma gondii* is an apicomplexan parasite responsible for toxoplasmosis. This parasitosis affects approximately one-third of the worldwide population [30]. Although benign in most cases, it can still pose risks to immunocompromized patients or fetuses [24, 30]. First-line therapy consists of the combination of pyrimethamine and sulfadiazine with leucovorin added to prevent hematologic toxicity. Atovaquone or azithromycin may be used as an alternate therapy in combination with pyrimethamine or sulfadiazine for the treatment and prophylaxis of toxoplasmosis when first-line therapy is contraindicated (possibility of serious side effects when pyrimethamine is associated with sulfadiazine). In addition, it was shown that the parasite can develop resistance to treatments [12, 29, 35].

Plants are an important natural reservoir of antimicrobial compounds [1]. In this context, the tree barks are starting material of interest as they protect the heartwood from insect, fungal or microorganism attacks. Black alder (*Alnus glutinosa*, Betulaceae) is a tree found throughout Europe. A decoction of the bark is used in some traditional medicines to fight against fever, mouth and pharyngeal inflammation, and rheumatism. This species is known for its richness in triterpene compounds such as betulin, betulinic acid, lupeol, and lupenone [15, 34]. Many studies have reported on the biological activity of triterpenes like antitumor, anti-inflammatory, hepatoprotective, gastroprotective, antimicrobial, anti-diabetic, and hemolytic activities [3, 5, 21, 42]. In apicomplexans and more particularly in *Plasmodium falciparum*, betulinic acid and its analogs disrupt the invasion process of the parasite *in vitro* [47]. Other betulinic acid derivatives have shown *in vivo* antiparasitic activity on two *Plasmodium* species [36]. Ursolic acid derivatives inhibit the *in vitro* activity of the PfHsp70-1 protein involved in the development of the parasite [37]. For *T. gondii*, lupane and ursane-type triterpenes from *Quercus crispula* have demonstrated *in vitro* antiparasitic activities [14]. Maslinic acid (oleanane triterpene) extracted from *Olea europaea* could also disrupt the host cell invasion process [10]. Nevertheless, the mechanism of action of triterpenes on *T. gondii* remains largely unknown. Only the docking of ursolic acid derivatives on the TgCDPK1 protein has been performed and has shown a strong affinity between the two entities [46], but the qualification of the interaction of triterpenes on other targets of *T. gondii* has not been investigated.

In a previous study, we demonstrated the strong anti-*T. gondii* activity of *n*-heptane bark extract of black alder (*Alnus glutinosa* (L.) Gaertn.) amongst 30 extracts of different polarities from 10 temperate tree species [9]. The phytochemical study of this extract revealed the majority presence of lupane type triterpenes. The first bioassays showed a correlation of the biological activity with the presence of triterpenes, especially betulone. An approach combining the determination of the IC_50_ and CC_50_ of the compounds of interest and an original inverse molecular docking workflow is proposed to better understand the mechanism of action of these molecules on *T. gondii.* Molecular docking aims to simulate the interaction between a ligand and a potential target [11]. Docking experimentation finds the best complexes of ligand/receptor according to the energy of binding. The crosslinking of these theoretical data with experimental data can further define the agonist effect of a small molecule toward a receptor. Inverse docking [44] allows for screening of a ligand on multiple receptors. In this way, it is possible to predict the preferred target of a ligand.

In this study, we present the purification of triterpenes of *Alnus glutinosa* bark extract and their activity on *T. gondii* through the determination of their IC_50_ values. An inverse docking process was then used to hypothesize the potential target of the most active compounds with the AMIDE reverse docking framework [8, 43]. The set of proteins was constructed using the Protein Data Bank (PDB) structures available on the Research Collaboratory for Structural Bioinformatics (RCSB) site (mid-2019) after elimination of redundant and low-quality models. Reverse docking was executed using the recently updated AMIDE framework [8, 43].

## Material and methods

### Chemicals and plant materials

Tree bark of *Alnus glutinosa* (L.) Gaertn. (Black alder, Betulaceae) was harvested in 2014 in the Champagne-Ardenne territory (northeast France) with authorization from the French “Office National des Forêts” (ONF). A voucher specimen (JH-2014-3) was deposited in the Herbarium of the Botanical laboratory at the faculty of Pharmacy of Reims (University of Reims Champagne-Ardenne, Reims, France). It was dried for 72 h and milled as described in a previous publication [18].

Betulonic acid (CAS: 4481-62-3) and betulonic acid methyl ester (CAS: 4356-31-4) were purchased from ClickBetulin (Ventspils, Latvia).

Acetonitrile (CH_3_CN), methanol (MeOH), methyl-*tert*-butyl ether (M*t*BE), and *n*-heptane were purchased from Carlo Erba Réactifs SDS (Val-de-Reuil, France). Deuterated chloroform (chloroform-d) was purchased from Sigma-Aldrich (Saint-Quentin, France).

### NMR analysis

Compounds to analyze were dissolved in 600 μL CDCl_3_. ^1^H, ^13^C, and 2D-NMR (HSQC, HMBC, and COSY) experiments were performed at 298 K on an Avance AVIII-600 (Bruker, Germany) spectrometer (^1^H at 600 MHz and ^13^C at 150 MHz) equipped with a 5 mm TCI cryoprobe. 2D-NMR experiments were performed using standard Bruker microprograms (TopSpin 3.2 software).

### Solid-liquid extraction and centrifugal partition chromatography fractionation

The dried and powdered *A. glutinosa* barks (100 g) were macerated in 1.5 L of *n*-heptane under stirring at room temperature overnight. The extract was then filtered and dried in a rotary evaporator, to give 1.1 g of extract (yield 1.1%). Extract fractionation was performed by Centrifugal Partition Chromatography (CPC) on a column with a capacity of 303.5 mL (FCPE300^®^, Rousselet–Robatel–Kromaton, Annonay, France) containing seven partition disks engraved with 231 twin partition cells (≈1 mL per twin cell). The CPC column was first filled by pumping the lower phase of the *n*-heptane/methanol (1:1, v/v) biphasic solvent system with 1% water at a flow rate of 50 mL/min with a KNAUER Preparative 1800 V7115 pump (Berlin, Germany) in the descending mode and a rotation speed of 500 rpm (27 g). Then, the CPC column was equilibrated by pumping the mobile phase (i.e., the upper phase of the same biphasic solvent system) in the ascending mode. The rotation was increased to 1200 rpm (158 g). Bark extract (1.2 g) solubilized in 20 mL of a mixture of stationary phase and mobile phase (8:2, v/v) was injected through a 3725 Rheodyne injector valve after solubilization. Then, 120 fractions were collected every minute using a Pharmacia Superfrac collector (Uppsala, Sweden). The separation was monitored by UV at 210, 254, 280, and 366 nm (UVD 170S detector, Dionex, Sunnyvale, CA, USA). All fractions were analyzed by thin layer chromatography (TLC) and then pooled according to their chromatographic profile, giving 20 chemically simplified fractions. TLC was performed on Merck 60 F254 pre-coated silica gel plates and developed with *n*-heptane/acetone (75:25 v/v). TLC plates were then sprayed with vanillin (5% w/v in EtOH) and H_2_SO_4_ solution (50% v/v in MeOH), followed by heating at 100–120 °C for 2–3 min.

### Semi-preparative HPLC purification

Terpene-rich fractions were solubilized in M*t*BE and then purified using a semi-preparative Uptisphere Strategy C18-HQ 250 × 10.0 mm (5 μm) column (Interchim, Montluçon, France) and a Dionex HPLC chain equipped with an ASI-100 autosampler, a P580 pump, a UVD 340S diode array detector, an automated fraction collector Ultimate 3000, and Chromeleon 6.8 software. Gradient elution with a constant flow rate of 5 mL·min^−1^ was used, starting with 90% of eluent A (CH_3_CN) and 10% of eluent B (H_2_O with 0.025% trifluoroacetic acid) reaching 100% of eluent A in 5 min, followed by isocratic elution with 100% of eluent A during 25 min. The effluent was monitored at 200 nm. Six triterpenes were purified: 4.7 mg of betulinic acid (**3**) (tr = 7.0 min), 31 mg of lupenone (**6**) (tr = 7.4 min), 20.9 mg of betulin (**1**) (tr = 8.7 min), 2.7 mg of betulone (**4**) (tr = 9.2 min), 11.7 mg of betulinaldehyde (**2**) (tr = 10.5 min), and 16.2 mg of lupeol (**5**) (tr = 11.0 min). A phytosterol was also purified, 7.2 mg of β-sitostenone (**9**) (tr = 7.8 min).

### Biological assays

#### Cell culture

Vero cells (host cells of *T. gondii* tachyzoite) were purchased at ATCC (reference CCL-81), grown in Iscove Modified Dulbecco Media (IMDM) GlutaMAX medium (Gibco) supplemented with 5% decomplemented fetal calf serum with 1% penicillin/streptomycin. The culture was incubated at 37 °C and 5% CO_2_. *Toxoplasma gondii* tachyzoites (strain RH, type I) were grown in the same conditions as Vero cells except that the concentration of fetal calf serum was 2%.

#### Stock solutions and dilutions

Stock solutions at 1 mg/mL were prepared in cell culture grade dimethyl sulfoxide (DMSO, PanReac AppliChem, Darmstadt, Germany). Subsequent dilutions were made in a culture medium identical to the one used for tachyzoite maintenance.

#### Cytotoxicity evaluation

The *in vitro* cytotoxicity was evaluated on Vero cell cultures using Uptiblue^®^ viable cell-counting assay (Interchim, Montluçon, France). CC_50_ assays were performed in 96-well plates containing 20,000 Vero cells and 200 μL of culture medium identical to the one used for Vero cell maintenance. After 4 h of incubation, wells were emptied. The IC_50_ was performed with 100 μL of solutions containing the tested compounds with a final concentration range of 0–80 μM. After 72 h of incubation, wells were washed with 100 μL of phosphate-buffered saline (PBS) (Sigma-Aldrich, Saint-Quentin-Fallavier, France). Then, 100 μL of IMDM culture medium supplemented with 2% (v/v) of calf fetal serum and 10% (v/v) of Uptiblue^®^ were added to each well. Plates were placed at 37 °C and 5% of CO_2_ for 3 h. Spectrophotometric measures were performed at 570 nm and corrected at 600 nm on an AMR-100 spectrophotometer (AllSheng, Hangzhou, China).

#### Screening at 10 μM and half-maximal inhibitory concentration (IC_50_)

All the experiments were performed in triplicate. Compound screening was performed at 10 μM in 96-well plates. Thus, 20,000 Vero cells contained in 200 μL of culture medium were filled in each well and left to adhere for 4 h at 37 °C and 5% of CO_2_. Then, 50,000 parasites contained in 50 μL of culture medium were filled, followed by 3 h of incubation in the same conditions. Wells were then emptied and 100 μL of solutions containing each drug at a final concentration of 10 μM were added. Incubation continued for 72 h. Parasite multiplications were revealed by enzyme immunoassay.

The IC_50_ assays were processed in the same conditions as previously described [40]. Dilutions were performed in a culture medium. The IC_50_ was determined using an adapted protocol from Doliwa *et al.* [12]. Parasites and cells were first fixed with 100 μL of cold methanol for 5 min at −20 °C. Cells were then rehydrated with 100 μL of PBS at room temperature. Wells were then emptied and filled with 60 μL of a solution of anti-*T. gondii* rabbit antibody (Ref. 9070-0556, Biorad) diluted at 1/1000. After 1 hour of incubation at 37 °C, wells were washed three times with PBS, and 60 μL of anti-rabbit IgG horseradish conjugated goat antibody (Ref. 170-6515, Bio-Rad) at 1/1000 were added, followed by 1 h of incubation at 37 °C. The wells were washed three more times, and 200 μL of a solution of o-phenylenediamine dihydrochloride (Sigma, France) was added. After 15 min at room temperature, the enzymatic reaction was stopped by the addition of 50 μL of hydrochloric acid (HCl) 3 M. To finish, 200 μL of each well were transferred to a new well and a spectrophotometer reading was performed at 492 nm, corrected at 630 nm. Each experiment was performed with three controls: control with host cells alone, control with host cells and parasites without the drug for parasite multiplication, and control with host cells with parasites and pyrimethamine at 1 μM.

#### Selectivity index

The value of the selectivity index (SI) was calculated for each compound according to the formula:

$$\mathrm{SI}=\frac{{\mathrm{CC}}_{50}}{{\mathrm{IC}}_{50}}.$$

### Statistical analysis of *in vitro* screening

Statistical analyses were performed using R software (version 4.1.1) with RStudio IDE (version 1.4.1717). Before pairwise comparison, data normality was assessed by a Shapiro test. Variance homogeneity between samples was then checked using a Fisher test before the pairwise comparisons of screening results with a classical Student *t*-test when both the normality and variance homogeneity null hypothesis were verified, or a Welsh two-sample *t*-test when only the null hypothesis about data normality was verified. Finally, a Wilcoxon rank-sum exact test was used for all other cases. Significance levels for *p*-values are: lower than 0.05 (*), lower than 0.01 (**), lower than 0.001 (***).

### Absorption and permeation computation

The SwissADME tool (http://www.swissadme.ch/) computes physicochemical descriptors of interest and predicts ADME parameters, pharmacokinetic properties and druglikeness nature of given compounds.

Thus, triterpenes descriptors related to the Lipinski compliance were computed (molecular weight, Moriguchi octanol-water partition coefficient MlogP, H-bond acceptors, H-bond donors) as well as the gastrointestinal (GI) absorption or blood-brain barrier (BBB) permeation (Table 1). Lipinski rules [20] suggest that poor absorption or poor permeation can be associated with a molecular weight higher than 500 Da, a MlogP higher than 4.15, to more than 10 H-bond acceptors, and to more than 5 H-bond donors. Only one violation of these rules is accepted. The BOILED-Egg model [7] estimates the GI absorption and the BBB permeation according to the lipophilicity and the polarity of the compounds.

**Table 1:** Absorption and permeation computation of the eight lupane-type triterpenes.

Compound	MW (Da)	MlogP	H-bond acceptors	H-bond donors	Lipinski compliant	GI absorption	BBB permeant
Betulin	442.72	6.00	2	2	Yes	Low	No
Betulinaldehyde	440.70	5.89	2	1	Yes	Low	No
Betulinic acid	456.70	5.82	3	2	Yes	Low	No
Betulone	440.70	5.89	2	1	Yes	Low	No
Lupeol	426.72	6.92	1	1	Yes	Low	No
Lupenone	424.70	6.82	1	0	Yes	Low	No
Betulonic acid	454.68	5.73	3	1	Yes	Low	No
Betulonic acid methyl ester	468.71	5.92	3	0	Yes	Low	No

### Inverse docking procedure

#### Computer hardware

All docking procedures were performed on Dell Optiplex 7920 equipped with Intel^®^ Xeon^®^ Silver 4216 2.1 GHz and NVidia Quadro RTX 4000 running under Ubuntu 20.04.

#### Ligand and protein preparation for inverse docking

Inverse docking was performed using the AMIDE v2 framework [8]. Its main characteristic is its speed of obtaining results. Each target (protein) is split into twelve boxes. The ligand is docked on each box and the calculations are distributed to several entities, such as CPU cores or GPUs.

Molecule 2D structures were drawn on ChemDraw 18.0 (https://perkinelmerinformatics.com/). The energy minimized 3D molecular structure of the docked compounds were then obtained with the LigPrep tool from Schrodinger suite (https://www.schrodinger.com/) with OPLS3e force field, ionization at pH 7 without tautomer generation. The resulting files were converted in PDBQT AutoDock readable format with Open Babel (https://openbabel.org/).

The structure of 218 proteins related to *T. gondii* were obtained from the Protein Data Bank (https://rcsb.org/) in mid-2019. Protein structures obtained by X-ray crystallography and corresponding to a resolution lower than 3 Å were selected. Thus, a final set of 87 non-redundant structures, including structures with co-crystallized co-factors was created. Then, each protein was submitted to AMIDE to be cut into 12 overlapping docking boxes. The number of AutoDock runs per box was set to 20.

#### General docking procedure

In a first step, betulone, the most *in vitro* active compound, was docked to the 87 proteins through the reverse docking process. The 10 first betulone/protein clusters obtained after the application of scorings 1 and 2 (described below) were selected and visually analyzed with PyMol 2 (https://pymol.org/2/) to discriminate plausible poses from unlikely poses. In particular, the presence of the ligand in a protein cavity and the existence of hydrogen bonds were verified. Finally, the selected poses of protein-ligand complexes were explored more thoroughly through new docking experiments focusing on a 40 × 40 × 40 Å^3^ box centered in the area of the best cluster. The parameters of these experiments were as follows: spacing equal to 0.375 Å, 150 runs using the Solis-Wets local search method combined with the use of AutoDock-GPU. Finally, the generated poses were also investigated with PyMol 2.

#### Inverse docking analyses and complements

After the AMIDE process, all protein-ligand complexes were submitted to a modified AutoDock Tools clustering script. The cut-off distance used in the clustering process was chosen based on the average value of the ligand radius of gyration. Each identified cluster was characterized by two parameters: its population and the best free energy of binding among its elements. For a given ligand/protein complex, the statistical character of the docking experiment was considered through the definition of a scoring function (scoring 1, Eq. (1)) that weighs energy and cluster population. Scoring 1 was used to re-rank the clusters of a considered ligand for each protein. Following the same idea, the diverse nature of the protein set was considered through the definition of a second scoring function (scoring 2, Eq. (2)) which weights the differences between the complexes. This second ranking was used to find betulone’s most promising target. For the two equations, Δ*G*_min_ was the lowest free energy of binding identified either within a given complex (scoring 1) or among all the complexes (scoring 2), Δ*G*_*x*_ was the free energy of binding of the considered cluster, Pop_max_ was the highest cluster population identified either within a given complex (scoring 1) or among all the complexes (scoring 2), and Pop_*x*_ was the population of the considered cluster. For equation (2), *Score* 1 _min_ was the score corresponding to the best complex among all the clusters, and *Score* 1_*x*_ was the score of the considered cluster. The lower the scoring, the better the result:

(1) $$\mathrm{Scoring}\;1\;=\;\sqrt{{\left(\frac{{\Delta G}_{\min}-{\Delta G}_{x}}{{\Delta G}_{\min}}\right)}^{2}+{\left(\frac{{\mathrm{Pop}}_{\max}-{\mathrm{Pop}}_{x}}{{\mathrm{Pop}}_{\max}}\right)}^{2}},$$

(2) $$\mathrm{Scoring}\;2=\;\sqrt{{\left(\frac{{\Delta G}_{\min}-{\Delta G}_{x}}{{\Delta G}_{\min}}\right)}^{2}+{\left(\frac{{\mathrm{Pop}}_{\max}-{\mathrm{Pop}}_{x}}{{\mathrm{Pop}}_{\max}}\right)}^{2}+{\left(\frac{{\mathrm{Score}\;1}_{\min}-{\mathrm{Score}\;1}_{x}}{{\mathrm{Score}\;1}_{\min}}\right)}^{2}}.$$

## Results

### Purification of the compounds of interest

**Figure 1:**
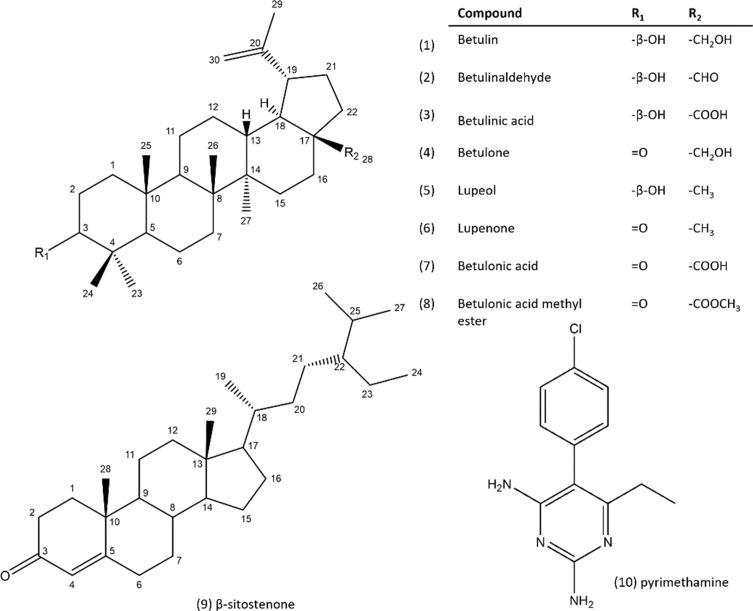
2D representation of purified compounds (**1–6**, **9**) from *n*-heptane extract of *A. glutinosa* bark, commercially available compounds (**7**, **8**), and reference antiparasitic compound (**10**).

The *n*-heptane bark extract of *Alnus glutinosa* was first fractionated by centrifugal partition chromatography and then, the triterpene-rich fractions were purified by semi-preparative reversed-phase HPLC. Seven compounds were purified and their structures were identified by NMR data analysis as 6 known lupane-type triterpenes: betulin (**1**) [25, 39], betulinaldehyde (**2**) [2, 25], betulinic acid (**3**) [25, 39], betulone (**4**) [26], lupeol (**5**) [25, 39], and lupenone (**6**) [4] together with the β-sitostenone (**9**), a phytosterol [32] (Fig. 1). The activity of the purified compounds (**1**–**5**, **9**) was then evaluated against *Toxoplasma gondii* except for lupenone (**6**) which was not soluble in DMSO.

### Screening at 10 μM

**Figure 2:**
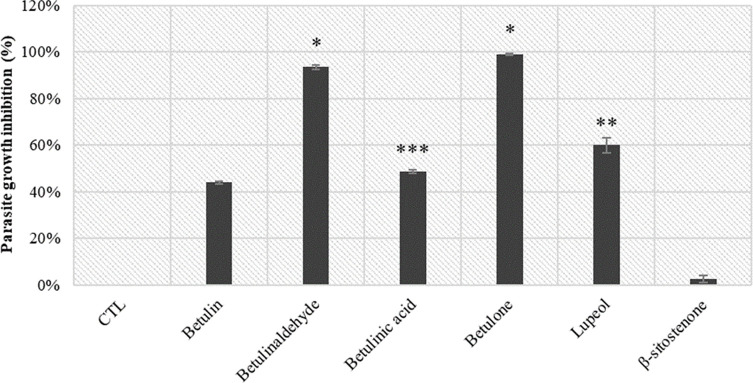
Screening at 10 μM of isolated compounds (**1–5**, **9**) from *n*-heptane bark extract of *A. glutinosa*.

Screening of the purified compounds **1**–**5** and **9** was performed at 10 μM (Fig. 2). The phytosterol β-sitostenone (**9**) isolated from *A. glutinosa* did not show any relevant activity. Betulinic acid (**3**) and betulin (**1**) showed 49% and 44% of parasite growth inhibition, respectively. Lupeol (**5**) had moderate action of 60%, but this result should be modulated due to the high standard deviation probably due to the low solubility of this triterpene. Betulinaldehyde (**2**) and betulone (**4**) showed the highest growth inhibition activity: 93% and 99%, respectively. Only lupane-type triterpenes **1–5** were thus kept for further studies. For comparison, the parasite growth inhibition of pyrimethamine (compound of reference) is 78% at 1 μM but the compound is cytotoxic at 10 μM so it was not used as a positive control at this concentration.

### Half maximal inhibitory concentration (IC_50_)

Table 2 shows the IC_50_ of the lupane-type triterpenes (**1–5**) as well as of the two commercially structurally related compounds betulonic acid (**7**) and betulonic acid methyl ester (**8**). Although the latter has never been described in *Alnus glutinosa* barks, they were nevertheless introduced into this study for structure-activity relationship considerations. Betulone (**4**) and betulonic acid methyl ester (**8**) showed the lower IC_50_ at 2.7 μM ± 1.2 and 3.1 μM ± 0.4, respectively. The IC_50_ of betulinaldehyde (**2**) and betulonic acid (**7**) was a little higher, with values of 5.1 μM ± 0.6 and 4.3 μM ± 1.4, respectively. Betulinic acid (**3**), betulin (**1**) and lupeol (**5**) had similar IC_50_ at 8.3 μM ± 2.7, 13.3 μM ± 7.3, and 13.8 μM ± 4.0, respectively. The standard deviation of results obtained for betulin (**1**) and lupeol (**5**) was high and could not be reduced because of difficulties to solubilize these compounds in medium culture. Regarding the results of bioassay at 10 μM, the IC_50_ of betulinic acid (**3**) should have theoretically been higher than 10 μM but this minor difference is inherent to the graphic determination of IC_50_.

**Table 2:** *In vitro* determination of IC_50_, CC_50,_ and SI from purified and commercial lupane-type triterpenes on *T. gondii*.

Compound	IC_50_ (μM)	CC_50_ (μM)	Selectivity index (SI)
Betulin (**1**)	13.3 ± 7.3	>80	>6.0
Betulinaldehyde (**2**)	5.1 ± 0.6	>80	>15.7
Betulinic acid (**3**)	8.3 ± 2.7	>80	>9.6
Betulone (**4**)	2.7 ± 1.2	>80	>29.6
Lupeol (**5**)	13.8 ± 4.0	>80	>5.8
Betulonic acid (**7**)	4.3 ± 1.4	>80	>18.6
Betulonic acid methyl ester (**8**)	3.1 ± 0.4	>80	>25.8
Pyrimethamine (**10**)*	0.7 ± 0.2	27.7	38.0

* Positive control.

### Cytotoxicity evaluation (CC_50_) and selectivity index

Table 2 highlights CC_50_ from 0 μM to 80 μM calculated for lupane-type triterpenes (**1–5**). All the CC_50_ were higher than 80 μM and a microscopic verification was performed for each well. Precise values could not be determined since solubility problems were encountered at concentrations higher than 80 μM. According to the results, betulone had a higher selectivity index (greater than 29.6).

### Absorption and permeation computation

In the light of the strong antiparasitic activity of some of these lupane-type triterpenes, it appears important to investigate the values of critical parameters related to their potential use as *in vivo* antiparasitics.

As a result, each of the eight compounds turned out to be Lipinski compliant since only their MlogP had a value higher than the limit given by the Lipinski rules. However, their ability to be absorbed by the gastrointestinal tract is low, and the simulation also suggests that they cannot cross the blood-brain barrier.

These data indicate that despite the high *in vitro* antiparasitic activity of the mentioned lupane-type triterpenes, a pharmacomodulation and/or formulation work on the most active compounds will have to be carried out to improve the absorption and permeation parameters while maintaining a satisfactory antiparasitic activity.

### Target identification of most active compounds by inverse docking

An inverse docking strategy using a dataset of *T. gondii* proteins obtained from the Protein Data Bank was performed to identify the potential biological targets of betulone, betulonic acid, betulonic acid methyl ester, and betulinic acid on *T. gondii*, and thus to obtain an initial suggestion of the mechanism of action. Betulone was first screened alone on the 87 proteins to identify the most promising proteins for the docking of the three other compounds. Table 3 presents data related to the 10 most relevant clusters identified from the docking of betulone; ranking is operated according to scoring 2 (Eq. (2)) which considers and compares cluster population, the free energy of binding, as well as scoring 1 (Eq. (1)).

**Table 3:** Cluster population, free energy of binding, scoring 1 and 2 of the 10 most relevant complexes containing betulone. The three underlined PDB IDs are selected proteins for re-docking experiments.

Protein	PDB ID	Population	Δ*G* (kcal/mol)	Scoring 1 (a.u.)	Scoring 2 (a.u.)
ENR	2O50	86	−8.42	0.6441	0.6577
DHFR	6AOI	95	−7.14	0.6247	0.6623
DJ-1	4XLL	74	−8.58	0.6917	0.7109
PLP1	6D7A	77	−7.23	0.6802	0.7334
OA	5EAV	68	−8.52	0.7167	0.7432
FBP A	5TKP	67	−8.28	0.7208	0.7532
CDPK3	3HZT	62	−9.44	0.7417	0.7658
ROP18	4JRN	61	−8.82	0.7461	0.7772
ROP8	3BYV	61	−8.63	0.7459	0.7798
MP 2	2XGG	65	−7.38	0.7318	0.7887

The values of scoring 2 spans between 0.6577 and 0.7887 and thus can be considered close. To narrow down the number of proteins used as targets in the re-docking experiments, a visual study of the 10 complexes was performed with PyMol 2 software. Betulone was selected as a reference since it showed the best *in vitro* activity. The best complexes were expected to present the ligand in a cavity and show electrostatic binding with the protein [38]. Proteins with PDB ID 2O50, 3HZT and 3BYV matched the requirements and were therefore selected. The protein with PDB ID 4JRN is equivalent to the PDB ID 3BYV in the defined region; in addition, the poses of the betulone best cluster were similar in the two structures. As a result, the protein with PDB ID 4JRN was not retained in the selection.

Based on previous results, the four most relevant compounds previously identified from *in vitro* experiments (betulone, betulonic acid, betulonic acid methyl ester, and betulinic acid) were submitted to docking simulations focused on the binding site previously identified for the three proteins. This refined docking was performed with 150 AutoDock runs to better qualify the poses. Results are presented in Table 4. The high population and low free energy of binding of the different complexes suggest strong binding between the four ligands and the three proteins. The additional AutoDock value of ligand efficiency was added to help in the selection of the best complex. All the compounds and proteins showed high affinity. The first hit was the complex betulonic acid methyl ester-3HZT with a ligand efficiency of −0.39, followed by the complex betulone-3HZT with a ligand efficiency of −0.36. The two cited complexes are described below as well as the first hit complex betulone-2O50 identified in Table 3.

**Table 4:** Population, free energy of binding, and ligand efficiency of the three re-docked ligand-complexes. Free energy of binding (Δ*G*) is expressed in kcal/mol. *POP* stands for cluster population. Ligand efficiency (*Lig. Eff.*) is an AutoDock Tools computed quantity. The lower the value, the better the complex.

Protein (PDB ID)		Betulone	Betulonic acid	Betulonic acid methyl ester	Betulinic acid
CDPK3 (3HZT)	POP	149	128	138	110
	Δ*G*	−11.52	−9.61	−12.74	−9.79
	Lig. Eff.	−0.36	−0.30	−0.39	−0.30
ENR (2O50)	POP	137	85	113	72
	Δ*G*	−9.55	−8.89	−10.87	−8.87
	Lig. Eff.	−0.30	−0.28	−0.33	−0.27
ROP8 (3BYV)	POP	150	150	139	111
	Δ*G*	−9.22	−7.34	−10.39	−7.74
	Lig. Eff.	−0.29	−0.23	−0.31	−0.23

**Figure 3:**
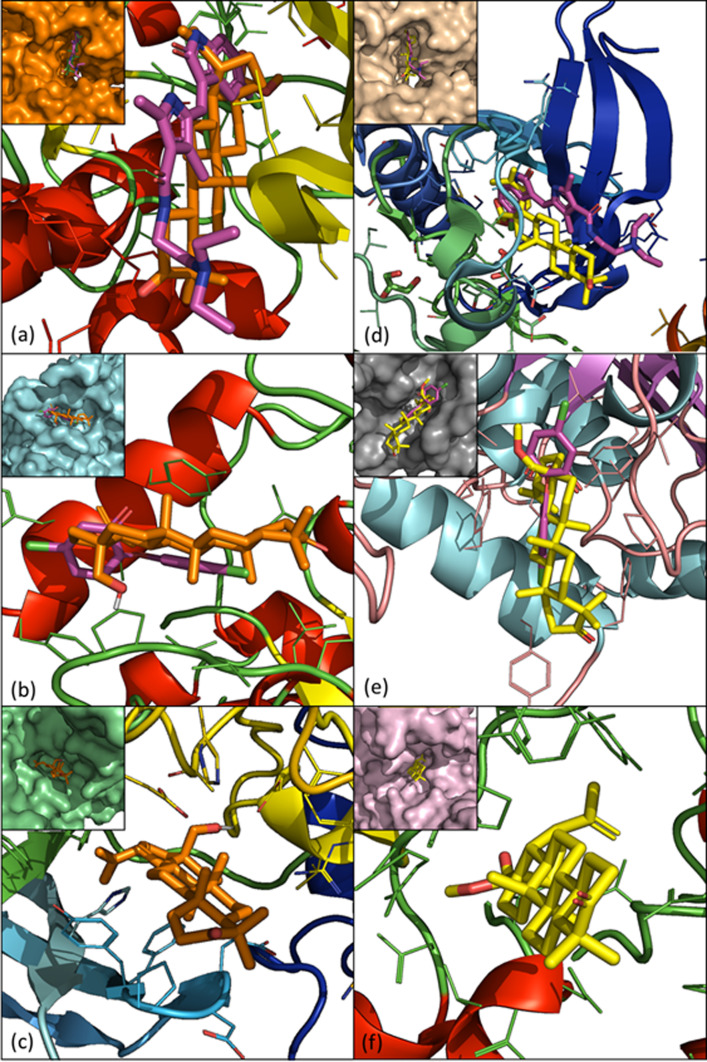
PyMol visualization of PDB ID 3HZT (CDPK3), 2O50 (ENR), and 3BYV (ROP8). (a) 3HZT with betulone (orange) and co-crystallized ligand (PDB ID J60, pink). (b) 2O50 with betulone (orange) and triclosan (pink). (c) 3BYV with betulone (orange). (d) 3HZT with BAME (yellow) and co-crystallized ligand (PDB ID J60, pink). (e) 2O50 with BAME (yellow) and triclosan (pink). (f) 3BYV with BAME (yellow). The inserts of each panel are the co-location of ligands and known inhibitors (if available) in the pockets (surfaces).

The best ligand efficiencies were obtained for the complexes with the ligands betulone and betulonic acid methyl ester (BAME) with the proteins with PDB IDs 3HZT, 2O50, and 3BYV. They were visualized with PyMol (Fig. 3). Each of the ligands was in a folded area that could be assimilated to a pocket (see inserts of Fig. 3). In addition, betulone and BAME were systematically in the binding site of original ligands for PDB IDs 3HZT and 2O50 which ensures that the ligands were docked to an area of interest. This comparison was not possible with PDB ID 3BYV since it does not have a co-crystallized ligand.

PyMol visualization of the BAME and PDB ID 3HZT protein complex shows that BAME was located in a pocket in which the co-crystallized ligand (PDB ID J60) is also located in the original 3HZT structure (Fig. 3d). Within 3 Å, the interaction analysis tool indicated polar contact between the ketone in position 3 and the residue GLU-160. Within 3.5 Å, the methyl ester in position 28 and the isopropyl group in position 19 of BAME seem to strongly interact with the residues of the cavity together with the CH_2_ and CH_3_ groups of the lupane backbone. This suggests strong interactions between BAME and the protein. These observations reinforce the conclusions drawn from the results shown in Table 4: there is a high affinity between BAME and the PDB ID 3HZT protein.

**Figure 4:**
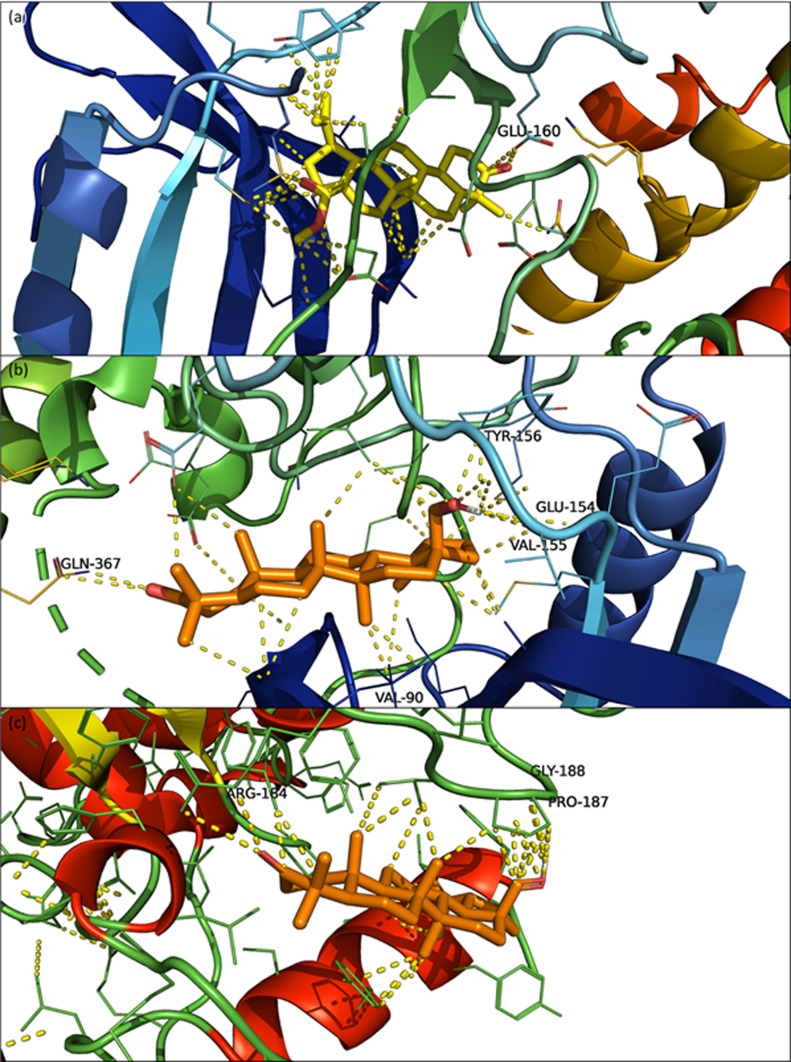
(a) PyMol visualization of the binding site of BAME (yellow) with PDB ID 3HZT (a); betulone (orange) in protein with PDB ID 3HZT (b); betulone (orange) with PDB ID 2O50 (c). Yellow dotted lines represent contacts between the complexes within 3.5 Å. Labeled residues associated with yellow dotted lines are contacts within 3 Å.

The complex betulone-3HZT showed the second-best free energy of binding equal to −11.52 kcal/mol and corresponded to a cluster population of 149. Visualization of the complex with PyMol showed that betulone was placed in the same cavity as BAME but was inverted lengthwise (Fig. 3a). Despite this, betulone still showed strong interactions with this receptor (Fig. 4b). Within 3 Å, polar contacts could be determined between the ketone in position C3 and GLN-367, and a polar area was defined between the C18 hydroxyl and residues GLU-154/VAL-155/TYR-156. Non-polar contact was established between the methyl group in position 27 with the residue VAL-90. Finally, many additional contacts were identified within 3.5 Å. Since the ligand can move within the cavity, these contacts provide a strong interaction between betulone and the protein.

Finally, the interaction between betulone and PDB ID 2O50 was studied since it was the first result obtained with the reverse docking process (Table 3). Within 3 Å, PyMol visualization of the complex betulone-2O50 (Fig. 4c) highlighted electrostatic interactions between residue ARG-184 and ketone in C3 of betulone and between residues PRO-187/GLY-188 and CH_2_–OH in position 28 of betulone. Within 3.5 Å, the lupane backbone seems to interact with the protein and a pi-stacking is even supposed with a tyrosine. Together with the backbone, the ketone and the CH_2_–OH functions seem to stabilize betulone in the cavity.

## Discussion

In a previous study [9], we demonstrated that the *n*-heptane extract of *Alnus glutinosa* bark had anti-*Toxoplasma gondii* activity. A bio-guided fractionation achieved by centrifugal partition chromatography and ^13^C NMR-based dereplication showed that the active fractions were mainly composed of lupane-type triterpenes. As a result, six triterpenes were isolated from these triterpene-rich fractions (betulin, betulinaldehyde, betulinic acid, betulone, lupeol, and lupenone) but only the five first compounds were tested. β-sitostenone was also isolated since it was a major constituent of the active fractions, but it revealed no specific activity. Their antiparasitic evaluation against *T. gondii* indicates that betulone showed the highest activity with an IC_50_ of 2.7 μM, while lupeol (**5**) had the highest IC_50_ at 13.8 μM. The selectivity indexes were all greater than 5.8, but since the compounds were not soluble at a higher concentration of 80 μM in the protocol, IS could not be precisely measured. Nevertheless, the CC_50_ from betulin on Vero cells was previously determined to be 164 μM [17]. Thus, in our context, the SI of betulone could be greater than 29. Only a few articles report the potential anti-*T. gondii* of triterpenes and most of them present lower activity than that obtained in this study. For example, maslinic acid – an oleanane-type triterpene – shows an LD_50_ of 53.8 μM for *T. gondii* [10] and an LD_50_ of 236.6 μM for Vero cells [10]. This suggests that lupane-type triterpenes have a higher anti-*T. gondii* potential than other triterpene types. This can be likened to conclusions reported by Gachet *et al.* highlighting that betulone (**2**) possesses interesting pharmacological activities especially on *Plasmodium falciparum* K1 strain, another apicomplexa, with an IC_50_ of 3.0 μM and SI of 7.4 [16].

Betulin and especially its oxo-derivatives (betulone, betulinic and betulonic aldehydes, and betulinic and betulonic acids) have valuable biologically active properties that were previously reported. For instance, betulinic acid and its derivatives exhibit anti-cancer, anti-HIV, antiviral, anti-inflammatory, antiseptic, antimicrobial, anti-malarial, anti-leishmaniasis, anthelmintic, and fungicidal activities, while betulonic acid shows pronounced anti-inflammatory, antimelanoma, and antiviral effects. The 3-oxo derivatives of betulin – betulone and its derivatives exhibit antitumor, anti-inflammatory, antiparasitic, and anti-HIV properties [31]. Domínguez-Carmona *et al.* showed that C3 hydroxyl group oxidation of betulin to produce betulonic acid resulted in a significant increase of leishmanicidal activity against *L. amazonensis* [13]. In our study, we observed that the presence of ketone and hydroxyl functions in C3 and C28, respectively, corresponding to betulone, enhanced the anti-*T. gondii* activity. Two commercial compounds, betulonic acid and betulonic acid methyl ester were then tested to validate this hypothesis and they showed IC_50_ of 4.3 μM and 3.1 μM, respectively without cytotoxicity at 80 μM on Vero cells. This confirmed that ketone on C3 improves lupane type triterpene ability to inhibit *T. gondii* growth. Da Cunha *et al.* showed that at the concentration of 50 μM, the inhibition of *L. donovani*, which was 35.0% and 39.8% with betulin and betulinic acid, respectively reached 97.6% with betulonic acid [6]. Isah *et al.* also showed structural modification especially at C3 and C27 of the parent backbone of pentacyclic triterpenes improved antiparasitic activity in some cases and loss of activity in others [19]. All these results confirm that ketone in C3 and hydroxyl or aldehyde on C28 improves the ability of lupane type triterpenes to inhibit parasite growth.

To investigate the mechanism of action of betulone on *T. gondii*, an inverse docking study of betulone on 87 proteins was carried out, highlighting a strong interaction between betulone and three *T. gondii* proteins identified as CDPK3 (PDB ID 3HZT), ENR (PDB ID 2O50), and a rhoptry domain kinase ROP8 (PDB ID 3BYV). These proteins are not found in the human body, which is interesting in terms of selectivity towards parasitic rather than human biological targets. CDPK3 is a calcium-dependent protein kinase involved in the specific calcium-signaling pathway in plants and apicomplexans. The inhibition of this protein could lead to the alteration of motility, cell invasion, and egress disruption [45]. There are only few publications describing the activity of pentacyclic triterpenes on CDPK. Nevertheless, one of them mentions that ursolic acid derivatives are active against *T. gondii in vivo* by inhibiting the proliferation of the parasite in the mouse abdominal cavity and in the same publication, Luan *et al.* identified CDPK1 (PDB ID 6BFA) as a potential target [23]. This protein is also involved in *T. gondii* egress regulation as well as CDPK3 [22]. These two results support the hypothesis that CDPK3 is a potential target of lupane type triterpenes.

Enoyl-acyl carrier protein reductases (ENR) are strongly involved in type II fatty acid synthesis and are considered essential for bacterial and protozoal survival. This is a target found in narrow-spectrum antibacterial drugs [28]. The prokaryotic-like type II fatty acid biosynthetic pathway in *T. gondii* is a validated molecular target in tachyzoites, it is essential for parasite survival *in vitro* and *in vivo* [27]. In particular, the enoyl reductase (ENR) enzyme, which catalyzes the last reductive step of the type II fatty acid synthesis pathway, is present in all *T. gondii* life cycle stages except microgametes and ENRs in other organisms may be the target for a wide range of potent inhibitors. Importantly, compounds that inhibit type II fatty acid synthesis (including triclosan and several newly designed and synthesized compounds) not only inhibit *T. gondii* tachyzoite growth, but are also effective against other apicomplexan parasites, such as *Plasmodium*. ENR is inhibited by the antibacterial compound triclosan which inhibits *in vitro* growth of *T. gondii* with IC_50_ of 3.0 μM [41]. Triclosan was found to inhibit *P. falciparum* growth with an IC_50_ of 1 μM [28]. The co-location of betulone and triclosan in the ENR binding site is consistent with the likely high affinity between betulone and ENR.

PDB ID 3BYV refers to rhoptry antigen kinase domain ROP8. The rhoptries can be released in the host cell in the internalization process. Even if the mechanism is not well known, these proteins could participate in a momentary breach in the cytoplasmic membrane of the host cell. According to Qiu *et al.*, this X-ray crystal structure was catalytically inactive but remains a template for the study of the major kinase ROP18 [33]. The interaction between pentacyclic triterpenes and ROP8 had never been reported before.

For the three proteins, there are many arguments that prove that the C3 oxidation level of betulone plays a major role in protein-ligand interactions, which can explain the *in vitro* results obtained in the framework of this work. Hydroxyl in C28 of betulone also interacts with amino acids of CDPK3 and ENR, but to date there is no evidence to support an interaction with ROP8.

In conclusion, this article highlighted the anti-*T. gondii* activity of lupane-type triterpenes isolated from black alder bark and the strong activity of betulone. Chemosensitivity of *T. gondii* to isolated triterpenes highlighted the importance of the ketone at the C3 position and the hydroxyl in C28. Reverse docking suggests that CDPK3, ENR, and ROP8 are potential targets for betulone, and interaction analysis confirmed the role of the functions in C3 and C28 deduced from preliminary structure-activity relationship studies. Further docking studies could be carried out on the three proteins with lupane-type triterpene derivatives that present a ketone in C3 and various functions in C28 to further the study of the anti-toxoplasmosis potential of lupane-type triterpenes. In addition, since these compounds cannot be correctly absorbed by the gastrointestinal tract or cross the blood-brain barrier, the candidates should be pharmacomodulated to improve these parameters before *in vivo* studies. In parallel, complementary studies should be carried out to develop galenic formulations including for instance micro-encapsulated triterpenes to overcome the solubility issues.

## Conflict of interest

The authors declare that they have no conflict of interest associated with this publication.
